# Epidemiology and treatment of atrial fibrillation in patients with type 2 diabetes in the UK, 2001–2016

**DOI:** 10.1038/s41598-020-69492-z

**Published:** 2020-07-27

**Authors:** Hassan Alwafi, Ian C. K. Wong, Amitava Banerjee, Pajaree Mongkhon, Cate Whittlesea, Abdallah Y. Naser, Wallis C. Y. Lau, Li Wei

**Affiliations:** 1 Research Department of Practice and Policy, School of Pharmacy, University College London, 29-39 Brunswick Square, London, WC1N 1AX UK; 2 Faculty of Medicine, Umm Al Qura University, Mecca, Saudi Arabia; 3 Department of Pharmacology and Pharmacy, Centre for Safe Medication Practice and Research, Li Ka Shing Faculty of Medicine, The University of Hong Kong, Pokfulam, Hong Kong; 4 Institute of Health Informatics, University College London, London, UK; 5 Barts Health NHS Trust, London, UK; 6 Division of Pharmacy Practice, Department of Pharmaceutical Care, School of Pharmaceutical Sciences, University of Phayao, Phayao, Thailand; 7 Pharmacoepidemiology and Statistics Research Center (PESRC), Faculty of Pharmacy, Chiang Mai University, Chiang Mai, Thailand; 8 Faculty of Pharmacy, Isra University, Amman, Jordan

**Keywords:** Arrhythmias, Diabetes, Epidemiology

## Abstract

Patients with Type 2 diabetes mellitus (T2DM) have an increased risk of atrial fibrillation (AF). The current study aimed to investigate the prevalence and treatment of AF in patients with T2DM, assess the impact of direct oral anticoagulants (DOACs) introduction on oral anticoagulant (OACs) prescribing rates, and factors associated with OAC initiations in patients with T2DM and AF. The Health Improvement Network (THIN) database (2001–2016), was used to examine the annual prevalence and treatment of AF in T2DM. The impact of DOACs introduction on OAC prescribing rates were investigated using interrupted time series analysis (ITS). Factors associated with OAC initiations were also identified using multivariate logistic regression. The prevalence of AF increased from 2.7 [95% confidence intervals (CI) 2.5–2.8] in 2001 to 5.0 (4.9–5.1) in 2016 per 100 persons. OACs prescribing within 30-days of AF diagnosis increased from 21.5% in 2001 to 56.8% in 2016. ITS analysis showed that OAC prescribing increased after DOAC introduction (*P* < 0.001), however, no immediate change was observed (*P* = 0.29). T2DM patients with AF, aged 60–79, male gender and BMI ≥ 25 were more likely to receive OAC, adjusted OR 1.3 (1.2–1.5) for aged 60–79, 1.3 (1.2–1.4) for male gender and 2.0 (1.9–2.2) for BMI ≥ 25, respectively. This study highlighted an increase in prevalence of AF in patients with T2DM during the study period. Further studies are warranted to investigate factors contributing to the underuse of OAC in patients with T2DM and AF.

## Introduction

Type 2 diabetes mellitus (T2DM) is one of the most common chronic diseases^1^. Patients with T2DM have an increased risk of comorbidities and mortality^1^. Atrial fibrillation (AF) is the most common form of arrhythmia, with about 1.6% of the population living with AF^2^. A meta-analysis of thirty-four studies reported that diabetes can increase the risk of AF by 28.0%^3^. Both T2DM and AF are independent risk factors for strokes and thromboembolic events^4^.

Patients with AF were mainly treated with warfarin for the prevention of stroke; however, studies have reported under prescribing with these medications^5^. In the last 15 years, important changes have occurred in the management of AF. This included the introduction of direct oral anticoagulants (DOACs) and the adoption of CHA_2_DS_2_-VASc scores, which includes diabetes as one of the important risk factors^6^. In addition, major guidelines now recommend using the CHA_2_DS_2_-VASc and DOACs as a first line therapy in the treatment of AF^6,7^. DOACs have a safer pharmacokinetic profile, fewer drug interactions, and less frequent monitoring in comparison to warfarin^8^, however, their effect on the rate of OACs prescribing remains unclear.

Previous studies that examined the prevalence and treatment of AF among patients with T2DM are limited. Estimating the burden of AF in patients with T2DM across the UK population will help to develop a better understanding of the co-existence of both conditions, their treatment and explore population levels trend in order to plan health policy.

The objectives of this study were (i) to examine the trends of the prevalence of AF in patients with T2DM from 2001 to 2016; (ii) to investigate the proportions of patients with T2DM who were initiated oral anticoagulants (OAC) on/or after AF diagnosis, and to assess the impact on OAC prescribing rates after the introduction of direct oral anticoagulants (DOACs), and (iii) to investigate factors associated with the initiation of OAC in patients with T2DM and AF.

## Methods

### Data sources

This retrospective population-based longitudinal study used data in the Health Improvement Network (THIN). THIN is a UK primary care database containing anonymized, clinical and prescribing data with more than 15 million cumulative patients, covering approximately 6.0% of the UK population^9^. THIN database is widely used healthcare database for the population-based medical research and has previously been used to study prescribing of OAC medications^10–14^.

### Ethical considerations

The present study is based on anonymised and unidentifiable THIN data, thus the need for informed consent was waived by the THIN scientific review committee (SRC). This study was reviewed and scientific approved by THIN SRC in 2018 (18THIN009). The research was reported in accordance with strengthening the reporting of observational studies in epidemiology (STROBE) Statement (Supplement).

### Study population

Patients with T2DM aged ≥ 18 years old and registered within THIN database between 2001 and 2016 were included in the study. Only patients who were registered with the general practice for at least 12 months prior to the first T2DM diagnosis being recorded were included. They were identified based on the Read Codes of (1) a diagnostic code for T2DM or (2) a non-specific code of diabetes but had a record of any prescribed oral hypoglycaemic agent. Patients who had a diagnostic code for T2DM accounted for 92.7% of the entire cohort, while the remaining were of criteria two. Patients who had a non-specific code of diabetes but had only records of insulin prescriptions were excluded because they may have type 1 diabetes mellitus (T1DM), although their age at first event was taken into account.

### Prevalence of AF in T2DM

T2DM patients who had a record of AF were identified on/or after their diagnosis of T2DM using the AF read codes, and the first record of AF was defined as the start date. Patients diagnosed with AF before the diagnosis of T2DM were excluded. Patients with valvular heart disease were excluded. Patients were censored if they left the practices, transferred out or died during the study period.

### OAC use in patients with T2DM and AF

**Figure 1:**
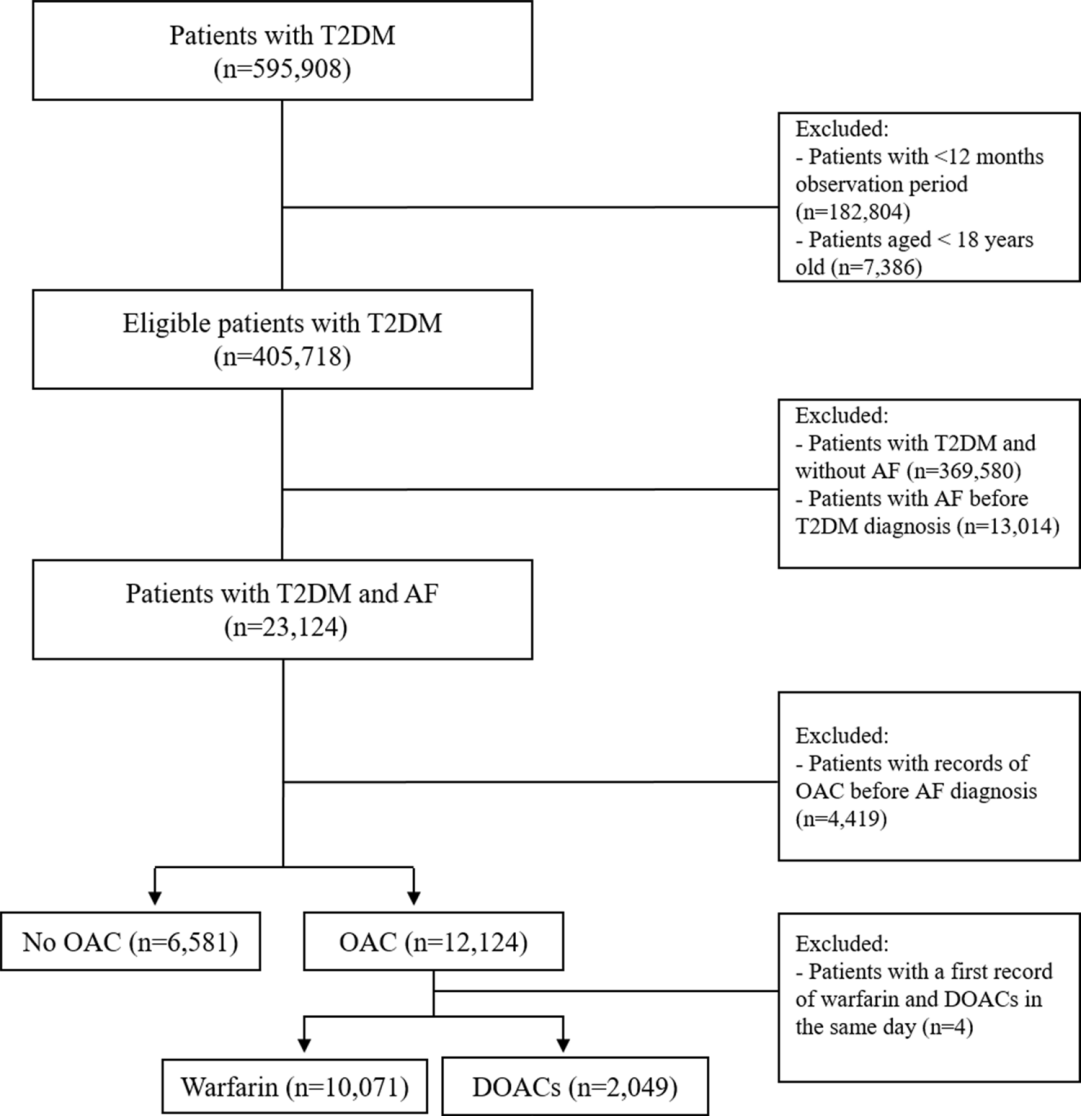
Selection of study population.

Patients with T2DM and AF who received at least one prescription of an OAC were identified using drug codes. Patients with T2DM who received an OAC prescription on/or after the diagnosis of AF were included in the treatment analysis. Patients who received an OAC prescription prior to the diagnosis of AF were excluded from the treatment analysis. Patients were divided into two groups; one group received an OAC prescription and a second group who did not receive an OAC prescription. Further stratification by type of OAC into warfarin and DOACs (dabigatran, apixaban, rivaroxaban, and edoxaban) were also undertaken. Selection of study cohort is presented in Fig. 1.

### Factors associated with OAC use

Patients characteristics included in the study were age, gender, smoking status, alcohol consumption, body mass index (BMI), coronary heart disease (CHD), heart failure, hypertension, hyperlipidaemia, chronic kidney disease (CKD), bleeding and liver diseases. These comorbidities were identified using Read codes^15,16^. Medications; antiplatelet drugs, antihypertensive drugs, and lipid-lowering drugs. In addition, we included polypharmacy as a covariate in the study and it was defined as the use of ≥ 4 chronic cardiac medications^17^. CHA_2_DS_2_-VASc score for stoke risk and HASBLED score for risk of bleeding were also calculated^6^.

### Outcomes

The primary outcome was the annual prevalence of AF in patients with T2DM from 2001 to 2016. Secondary outcomes were: the proportions of patients with T2DM who were initiated an OAC on/or after AF diagnosis from 2001 to 2016; the effect of the introduction of DOACs on the rate of OACs initiations in patients with T2DM and AF; and factors associated with OAC initiation in patients with T2DM and AF.

### Data analysis

Descriptive statistics were used to describe patients’ demographics, medications use and comorbidities. Continuous data were reported as mean ± standard deviation (SD), and categorical data were reported as number (percentage). The prevalence of AF in patients with T2DM was presented per 100 person with 95% confidence intervals (CIs). This was calculated annually by dividing the number of all T2DM patients diagnosed with AF during the particular year over the mid-year population of patients with T2DM in the same calendar year during the study period. Trends in the prevalence of AF were further stratified by age and gender. Temporal trends in the distribution of the prevalence were assessed using a Poisson method. The annual proportions of patients with T2DM who initiated an OAC (PPIOAC) on/or after AF diagnosis from 2001 to 2016 was calculated.

$$ PPIOAC = \frac{Number \;of\; patients\; with\; T2DM \;who\; received \;OAC \;on/or\; after\; the\; diagnosis \;of\; AF\; in\; a\; particular\; year }{{Number \;of\; patients \;with \;T2DM \;and \;were \;diagnosed \;with \;AF\; in\; that \;particualr \;year }} $$

Only patients who received OAC prescriptions within 30-days of AF diagnosis were accounted as OAC users (received OAC). However, we also conducted sensitivity analysis by accounting for patients who received OAC prescriptions within 90-days and within 1-year of AF diagnosis.

The impact of the introduction of DOACs on the rate of OAC initiation was plotted graphically over time. In addition, we fitted a segmented regression analysis using a Poisson regression^18^. Durbin-Watson test was used to examine any first order autocorrelation that may lead to an overestimations of the significance of an intervention. Residual analyses were conducted, and showed no evidence of autocorrelations. Overall, we included 44 data points (monthly quarters); representing repeated OAC prescriptions from July–October 2005 up to April-July 2016. DOACs were first authorized for the treatment of non-valvular AF in 2011^19^, therefore, we accounted for the intervention in this model from the first quarter of the next calendar year (January–April 2012). We used multivariable logistic regression to identify factors associated with the initiation of OACs prescribing in patients with T2DM and AF compared with no OAC prescribing, and stratified by OAC type (warfarin Vs. DOACs). Unadjusted and adjusted odds ratios (OR) with 95% confidence intervals (CIs) were estimated for all the aforementioned baseline covariates. All analyses were performed using SAS version 9.4 (SAS Institute, Cary, NC, USA).

## Results

### Demographics and characteristics

During the study period of 2001 and 2016, a total of 405,718 patients with T2DM were identified of whom only 23,124 patients with T2DM and AF were included. Around 12,124 (52.4%) received an OAC prescription at some point on/or after the diagnosis of AF (Fig. 1). The characteristics of patients are summarised in (Table 1).

**Table 1:** Baseline characteristics of patients with T2DM and AF.

Characteristic	OAC versus non-OAC			Warfarin versus DOACs		
	OAC** (n = 12,124)	No OAC (n = 6,581)	*p* value	warfarin (n = 10,071)	DOAC (n = 2,049)	*p* value
Age (Mean ± SD)*	73.4 ± 9.1	77.5 ± 10.5	< 0.001	73.6 ± 8.9	75.2 ± 9.6	< .0001
Gender (Male)	7,446 (61.4)	3,456 (52.5)	< 0.001	6,231 (61.8)	1,213 (59.2)	0.073
Smoking			< .0001			0.004
Never-smoker	5,450 (45.3)	3,099 (47,9)		4,443 (44.5)	999 (48.8)	
Ex-smoker	5,445 (45.3)	2,650 (40.9)		4,692 (47.0)	871 (42.5)	
Current-smoker	1,121 (9.3)	717 (11.0)		855 (8.5)	179 (8.7)	
Alcohol			< .0001			< .0001
Never-drinker	2,778 (23.9)	1,831 (29.8)		2,287 (23.7)	549 (27.5)	
Ex-drinker	604 (5.2)	323 (5.3)		509 (5.2)	130 (6.5)	
Current-drinker	8,235 (70.8)	3,976 (64.8)		6,875 (71.1)	1,319 (66.0)	
BMI			< .0001			0.036
BMI < 25	1,503 (12.5)	1,525 (24.2)		1,213 (12.2)	292 (14.3)	
BMI 25–30	3,891 (32.6)	2,181 (34.6)		3,239 (32.7)	655 (32.1)	
BMI ≥ 30	6,544 (54.8)	2,581 (41.0)		5,454 (55.1)	1,089 (53.4)	
CHA_2_DS_2_-VASc Score			< .0001			0.224
CHA_2_DS_2_-VASc Score < 2	279 (2.3)	151 (2.3)		206 (2.0)	37 (1.8)	
CHA_2_DS_2_-VASc Score ≥ 2	11,845 (97.7)	6,430 (97.7)		9,865 (98.0)	2,012 (98.2)	
HASBLED			0.862			0.252
HASBLED < 2	517 (4.2)	340 (5.2)		355 (3.5)	77 (3.8)	
HASBLED ≥ 2	11,670 (95.7)	6,241 (94.8))		9,716 (96.5)	1,972 (96.2)	
Coronary heart disease	4,052 (33.4)	2,216 (33.6)	0.3398	3,511 (34.8)	665 (32.4)	0.035
Heart failure	1,885 (15.5)	1,237 (18.8)	< .0001	1,959 (19.4)	342 (16.7)	0.009
Hypertension	9,365 (77.2)	4,813 (73.1)	< .0001	7,829 (77.7)	1,620 (79.0)	0.297
Hyperlipidaemia	3,085 (25.4)	1,440 (21.9)	< .0001	2,595 (25.7)	544 (26.5)	0.625
Stroke/TIA	2,094 (17.3)	1,257 (19.1)	0.0058	1,957 (19.4)	451 (22.0)	0.010
Bleeding	2,511 (20.7)	1,448 (22.0)	0.0064	2,114 (21.0)	516 (25.2)	0.006
Chronic Kidney Disease	3,644 (30.0)	2,101 (31.9)	0.0011	3,225 (32.0)	750 (36.6)	0.000
Aspirin	7,369 (60.8)	4,058 (61.7)	0.3477	6,568 (65.2)	1,123 (54.8)	< .0001
ACEs /ARBs	8,843 (72.9)	4,159 (63.2)	< .0001	7,809 (77.5)	1,515 (74.0)	< .0001
Beta-Blockers	5,882 (48.5)	2,515 (38.2)	< .0001	6,127 (60.9)	1,423 (69.4)	< .0001
Calcium Channel Blockers	5,525 (45.6)	2,508 (38.1)	< .0001	4,687 (46.5)	915 (44.6)	0.108
Statins	8,904 (73.4)	4,000 (60.8)	< .0001	7,693 (76.3)	1.598 (78.0)	0.343
Polypharmacy	3,324 (27.4)	1,542 (23.4)	< .0001	3,983 (39.5)	732 (35.7)	< .0001

### Prevalence of AF in patients with T2DM

**Figure 2:**
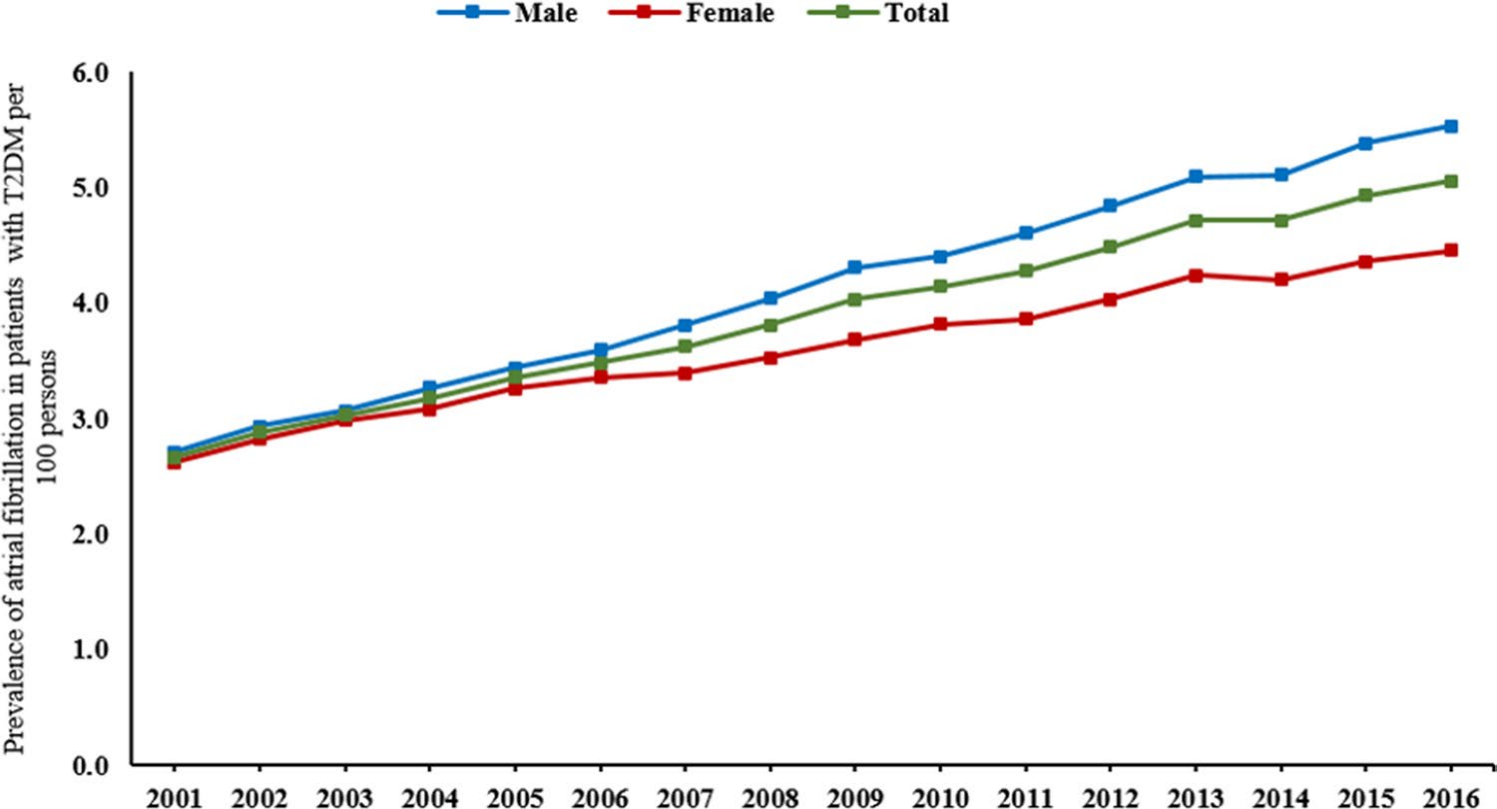
Prevalence of atrial fibrillation in patients with T2DM stratified by gender.

The prevalence of AF in patients with T2DM increased from 2.7% (95% CI 2.6–2.8), 2.7% (95% CI 2.5–2.9) for men and 2.6% (95% CI 2.4–2.8) for women in 2001 to 5.0% (95% CI 4.9–5.1), 5.5% (95% CI 5.4–5.6) for men, *p* < 0.001 and 4.4% (95% CI 4.3–4.6) for women in 2016 per 100 persons with T2DM, *p* < 0.001 (Fig. 2). Similarly increased trends for both men and women were observed for the first two years and then men started to have a higher increase rate over the study period than women (Fig. 2).

**Figure 3:**
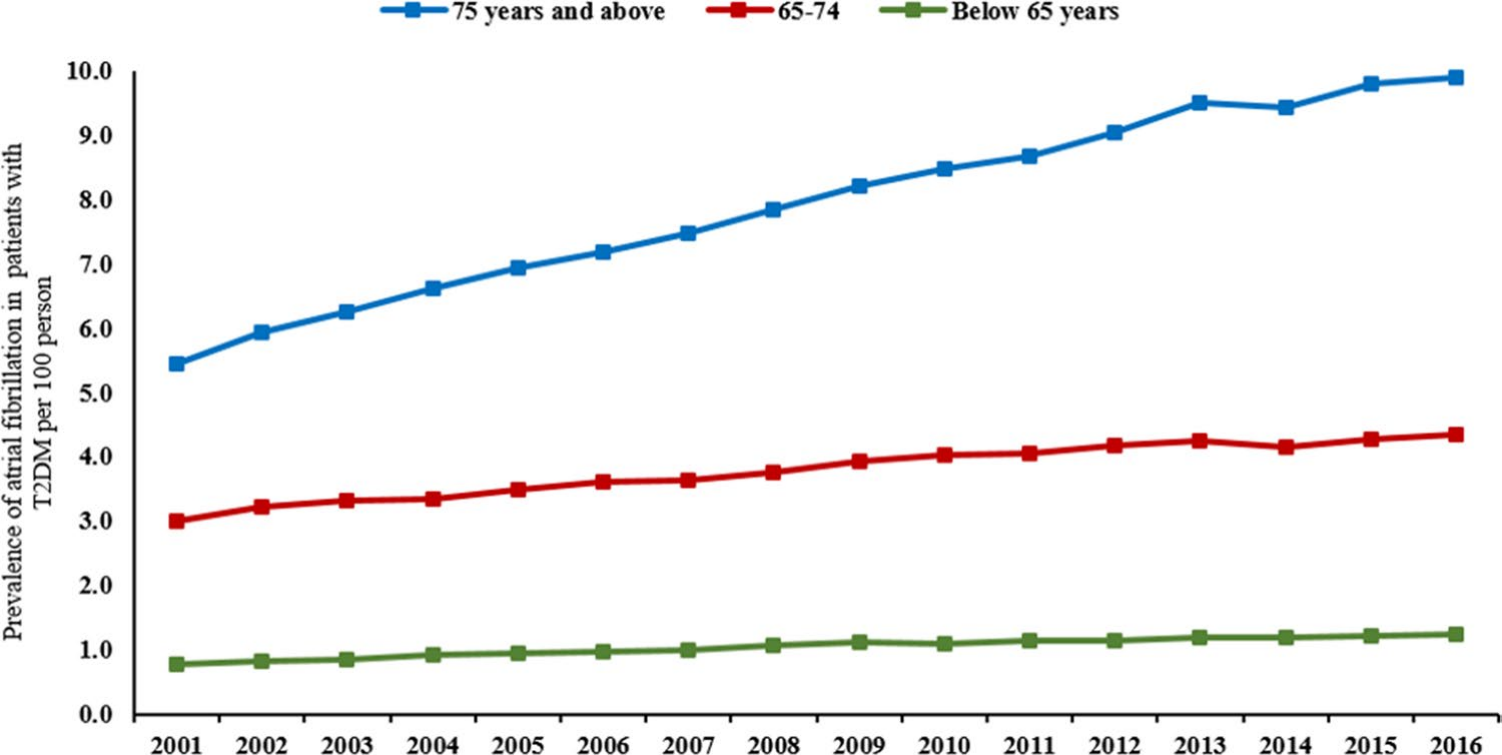
Prevalence rate of atrial fibrillation in patients with T2DM stratified by age.

The prevalence of AF varied among the different age groups. The prevalence of AF among patients aged 75 years and above increased from 5.5% (95% CI 5.1–5.8) in 2001 to 9.9% (95% CI 9.7–10.0) in 2016 per 100 persons with T2DM, *p* < 0.001. There was about 43–55% increase in AF prevalence among younger patients from 3.0% (95% CI 2.7–3.2) in 2001 to 4.3% (95% CI 4.2–4.4) in 2016 per 100 persons with T2DM, p < 0.001, for patients aged between 65 and 74 years, from 0.8% (95% CI 0.7– 0.9) in 2001 to 1.2% (95% CI 1.2–1.3) in 2016 per 100 persons with T2DM, *p* < 0.001, for patients aged below 65 years (Fig. 3).

### OAC treatment at various time points after AF diagnosis

**Figure 4:**
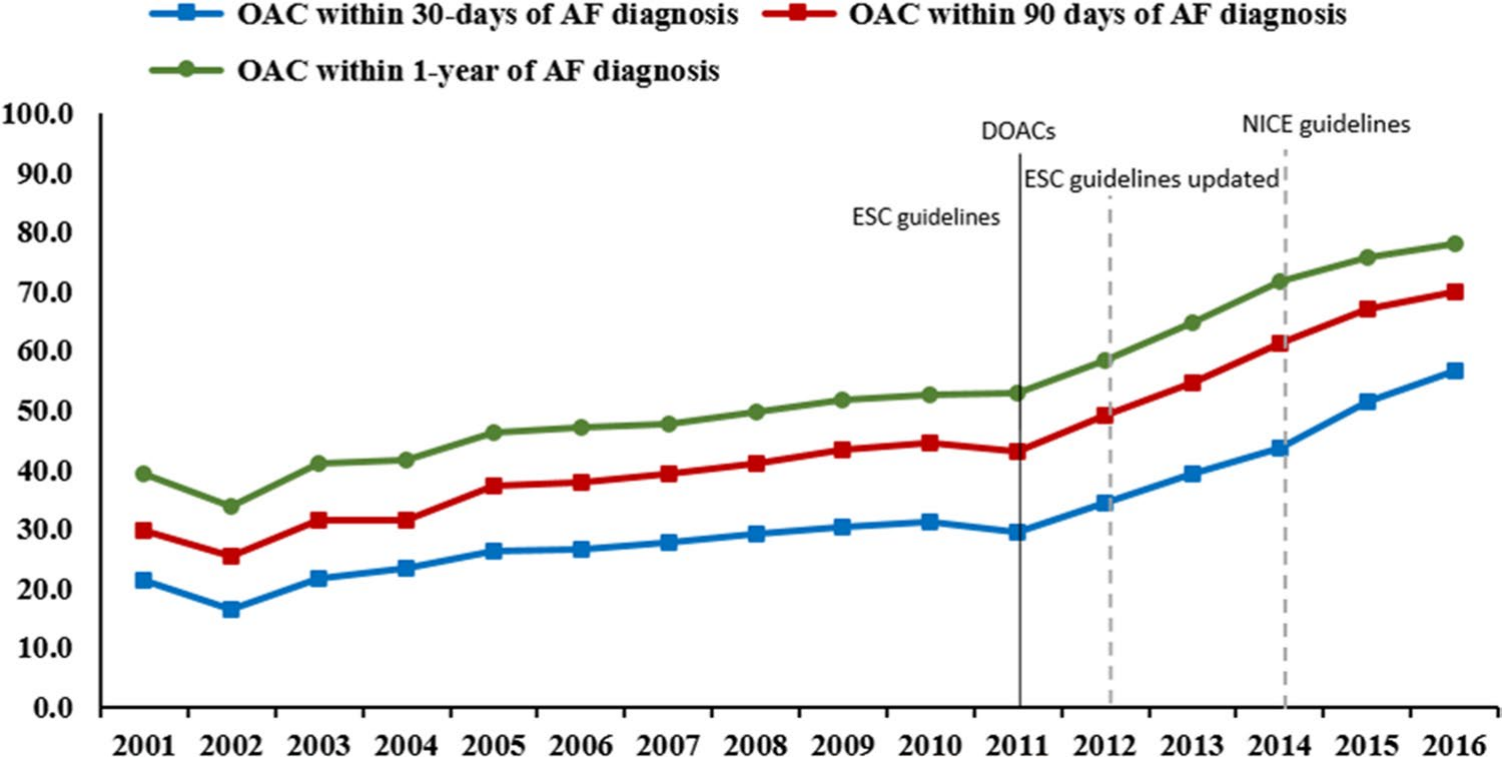
Proportion of T2DM patients who initiated OAC treatment after the diagnosis of AF, 2001–2016.

The proportions of patients with T2DM who received an OAC prescription within 30-days of AF diagnosis increased from 21.5% in 2001 to 56.8% in 2016, *p* < 0.001. In sensitivity analysis, the proportions of patients with T2DM who received an OAC prescription within 90-days of AF diagnosis was higher, 29.8% in 2001 to 69.9% in 2016, *p* < 0.001. In addition, the proportions of patients with T2DM who received an OAC prescription within 1-year after the diagnosis of AF was markedly higher in comparison to 30-days and 90-days from diagnosis, ranging from 39.4% in 2001 to 78.0% 2016, *p* < 0.001 (Fig. 4).

### Effect of the introduction of DOACs on OAC prescribing

**Figure 5:**
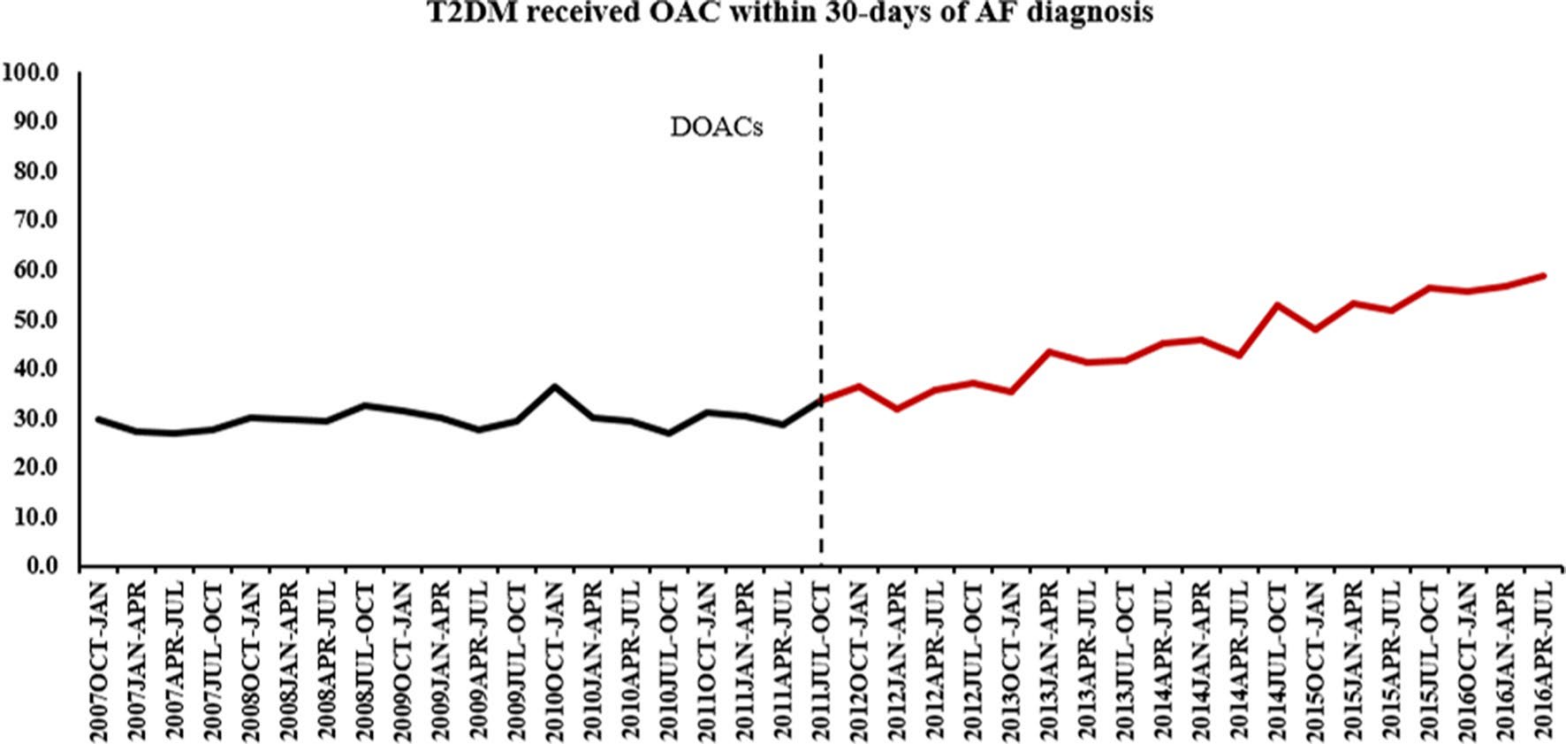
Monthly proportions of patients with T2DM who received OAC prescription after 30-days of AF, 2001–2016.

The overall monthly proportions of patients with T2DM who received OAC prescription on/or after 30-days of AF is presented in (Fig. 5). There was no immediate change in the rate of OAC prescribing after the introduction of DOACs (*p* = 0.29). However, the rate of OAC initiation then increased gradually (*p* < 0.001) (Table S1).

### Factors associated with initiation of OAC prescription versus non-OAC

In the multivariable logistic regression analysis, males patients were 30.0% more likely to initiate OAC compared to females adjusted OR 1.3; (95% CI 1.2–1.4). Patients aged 65–74 were more likely to receive OAC prescription adjusted OR 1.3; (95% CI 1.2–1.5) compared to patients younger than 65 years, while elderly patients aged ≥ 75 were less likely to receive OAC prescription adjusted OR 0.8;(95% CI 0.7–0.9). BMI ratios (BMI 25–29 and BMI ≥ 30) were significantly associated with OAC initiation compared to BMI < 25 adjusted OR 1.6; (95% CI 1.4–1.7) and adjusted OR 2.0; (95% CI 1.9–2.2), respectively). In addition, the use of ACEI/ARB, BB, CCBs and statins was a strong predictor to initiate OAC, while use of aspirin and polypharmacy were protective factors against the initiation of OAC. Table 2 presents details of the results from a logistic regression model.

**Table 2:** Factors associated with OAC initiation in patients with T2DM and AF.

Variables	OAC versus non-OAC				Warfarin versus DOACs			
	Unadjusted OR (95% CI)	*p* value	Adjusted OR (95% CI)	*p* value	Unadjusted OR (95% CI)	*p* value	Adjusted OR (95% CI)	*p* value
Age < 65	Reference		Reference		Reference		Reference	
Age 65–74	1.2 (1.1–1.4)	0.000	1.3 (1.2- 1.5)	< .0001	0.9 (0.8–1.1)	0.491	0.8 (0.7–1.0)	0.081
Age ≥ 75	0.6 (0.5- 0.6)	< .0001	0.8 (0.7- 0.9)	< .0001	0.7 (0.6–0.8)	< .0001	0.6 (0.5–0.8)	< .0001
Male sex (%)	1.40 (1.3–1.5)	< .0001	1.3 (1.2–1.4)	< .0001	1.1 (1.0–1.2)	0.073	0.9 (0.8–1.0)	0.038
Never-smoked	Reference		Reference		Reference		Reference	
Ex-smoker	1.1 (1.0–1.2)	< .0001	1.0 (0.9–1.1)	0.914	1.2 (1.1–1.3)	0.001	1.1 (1.0–1.3)	0.019
Current-smoker	0.9 (0.8–1.0)	0.012	0.8 (0.7–0.9)	< .0001	1.0 (0.8–1.3)	0.588	0.9 (0.8–1.1)	0.459
Never-drink	Reference		Reference		Reference		Reference	
Ex-drinker	1.2 (1.1–1.4)	0.006	1.0 (0.9–1.2)	0.354	0.9 (0.8–1.2)	0.618	0.9 (0.7–1.1)	0.432
Current-drinker	1.3 (1.3–1.4)	< .0001	1.2 (1.1–1.3)	< .0001	1.3 (1.1–1.4)	< .0001	1.2 (1.0–1.3)	0.003
BMI < 25	Reference		Reference		Reference		Reference	
BMI 25–29	1.8 (1.7–2.0)	< .0001	1.6 (1.4–1.7)	< .0001	1.2 (1.0–1.4)	0.025	1.1 (0.9- 1.3)	0.204
BMI ≥ 30	2.6 (2.4–2.8)	< .0001	2.0 (1.9–2.2)	< .0001	1.2 (1.0–1.4)	0.012	1.0 (0.9 1.2)	0.720
CHA_2_DS_2_-VASc Score < 2	Reference		Reference		Reference		Reference	
CHA_2_DS_2_-VASc Score ≥ 2	1.0 (0.8–1.2)	0.863	1.1 (0.8–1.4)	0.385	0.9 (0.6–1.3)	0.695	1.0 (0.7–1.6)	0.863
HASBLED < 2	Reference		Reference		Reference		Reference	
HASBLED ≥ 2	1.1 (0.9–1.3)	0.367	0.9 (0.8–1.2)	0.726	1.2 (0.9–1.5)	0.241	1.2 (0.8- 1.6)	0.361
Coronary heart disease	1.0 (0.9–1.0)	0.339	0.9 (0.8–0.9)	0.000	1.1 (1.0–1.3)	0.035	1.1 (0.9- 1.2)	0.337
Heart Failure	0.8 (0.7–0.9)	< .0001	0.9 (0.8–0.9)	0.001	1.2 (1.0–1.3)	0.009	1.2 (1.0–1.3)	0.025
Hypertension	1.2 (1.1–1.3)	< .0001	0.9 (0.9–1.0)	0.160	0.9 (0.8–1.0)	0.297	0.9 (0.8–1.1)	0.331
Hyperlipidaemia	1.2 (1.1–1.3)	< .0001	1.0 (1.0–1.1)	0.224	1.0 (0.9–1.1)	0.624	1.0 (0.9–1.1)	0.605
Stroke/TIA	0.9 (0.8–1.0)	0.006	1.0 (0.9–1.1)	0.621	0.9 (0.7–1.0)	0.010	0.9 (0.8–1.0)	0.045
Bleeding	0.9 (0.8–1.0)	0.010	0.9 (0.8–1.0)	0.029	0.8 (0.7–0.8)	< .0001	0.8 (0.7–0.9)	0.000
Chronic Kidney Disease	0.9 (0.8–1.0)	0.001	0.9 (0.9–1.0)	0.205	0.8 (0.7–0.9)	.0008	0.9 (0.8–1.0)	0.041
Aspirin	1.0 (0.9–1.1)	0.334	0.9 (0.8–0.9)	< .0001	1.5 (1.4–1.7)	< .0001	1.5 (1.4–1.7)	< .0001
ACEI/ARB	1.5 (1.4 1.6)	< .0001	1.3 (1.2–1.4)	< .0001	1.2 (1.1–1.4)	< .0001	1.2 (1.0–1.3)	0.019
Beta-blockers	1.5 (1.4–1.6)	< .0001	1.5 (1.4- 1.6)	< .0001	0.7 (0.6–7.0)	< .0001	0.6 (0.5–0.7)	< .0001
Calcium Channel Blockers	1.3 (1.2–1.4)	< .0001	1.3 (1.2- 1.4)	< .0001	1.0 (1.0–1.2)	0.109	1.0 (0.9–1.1)	0.884
Statin	1.7 (1.6–1.8)	< .0001	1.5 (1.4 1.6)	< .0001	0.9 (0.8–1.0)	0.343	0.8 (0.7- 1.0)	0.011
Polypharmacy	1.2 (1.1–1.3)	< .0001	0.8 (0.7–0.9)	< .0001	0.8 (0.8–1.0)	0.000	1.2 (1.0–1.3)	0.015

### Factors associated with initiation of warfarin versus DOACs

T2DM patients with AF aged ≥ 75 years adjusted OR 0.7 (95% CI 0.6–0.8) were more likely to be prescribed DOACs compared with patients age under 65 years old. T2DM patients with AF who had a history of using aspirin and angiotensin converting enzyme inhibitors (ACEs/ARBs) were significantly associated with higher odds of initiating warfarin adjusted OR 1.5 (95% CI 1.4–1.7) and 1.1 (95% CI 1.0–1.3), respectively. In contrast, having a history of bleeding 0.8 (95% CI 0.7–0.9), CKD 0.9 (95% CI 0.8–0.9), or history of using beta-blockers (BB) 0.6 (95% CI 0.5–0.7) were significantly associated with higher odds of initiating DOACs (Table2).

## Discussion

In this population-based study, we investigated trend in the prevalence and treatment of AF in patients with T2DM over a 16-year period. The key findings were: 1) the prevalence of AF in patients with T2DM has increased from 2001 to 2016, 2) the proportion of patients with T2DM who were initiated on an OAC after AF diagnosis increased between 2001 and 2016, 3) the rate of OAC initiation after the introduction of DOACs into the market increased, and 4) our study demonstrated that age ≥ 75 years, previous bleeding or stroke/TIA and history of CKD, were strong predictors for DOACs initiation.

Previous studies reporting the prevalence of AF in patients with T2DM are lacking, A study by Adderley et al., using a national UK database, reported that the prevalence of AF in the UK general population increased from 2.0% in 2000 to 3.2% in 2016^20^. The authors reported that the prevalence of AF was higher among those aged 65 years and above and was higher among male patients which was supported by our study findings in the T2DM patients. Our results showed a higher prevalence trend among male patients and among those aged 65 years and above which was similar to their results. In addition, the increase of AF prevalence in patients with T2DM over the years could also be related to an evolved physician's sensibility and consequent more aggressive search for AF^21^.

Ageing is an important risk factor for AF and the prevalence of AF increases with age, in the Framingham study it was reported that the prevalence of AF increased by 0.5% for those aged 50–59 years compared to 8.8% for those aged 80–89 years^22^. Furthermore, in a European community based studies it was reported that cumulative incidence of AF increased markedly after the age of 50 for men and 60 for women^23^. Our study showed a higher trend in the prevalence of AF in males and patients aged 75 years and above compared to females and patients younger than 75 years, which was similar to published data for the general population^22,23^.

Previous studies reported the association of T2DM and AF; however, the mechanism of the development of AF in patients with T2DM is not fully understood. It has been suggested that the metabolic process in patients with T2DM, including the inflammatory response and the atrial remodelling, might play a major role in the association between both diseases^24,25^. In addition, patients with T2DM have a high cardiac risk-profile and a higher body mass index. These are known risk factors for AF, which was highlighted in our study^26,27^.

T2DM and AF are both highly prevalent in the general population with about 6.0%-7.0% of the population having diabetes^1^ and about 1.5%–2.9% of the population have AF^2,20^. T2DM and AF have also been linked to several comorbidities and increased risk of stroke and mortality. It is therefore important to recognise the coexistence of both conditions to increase the awareness and to closely monitor this population. Several guidelines rely on the CHA2DS2–VASc score, in which T2DM is a criterion for score calculation and have recommended the use of OACs in patients with atrial fibrillation in order to prevent future stoke events^6,7^.

Although, our study demonstrated that the rate of OAC initiation has increased over time, our study also highlighted the possible underuse of OAC in this population. Particularly if we take into consideration that the majority of the patients were eligible for anticoagulation, based on the CHA_2_DS_2_–VASc score, as shown in Table 1^6^. There were 44.0% of our study patients in 2016 who still did not receive OAC within 30-days of AF diagnosis. It is important to mention there are other factors that physicians might consider before prescribing an OAC to their patients including risk of bleeding^6,7,28^.

In this study, we found that the rate of OAC initiation after the introduction of DOACs increased significantly, however, this change was not immediate. This could be explained because new drugs are prescribed with a greater caution due to uncertainties in regards to their effectiveness and safety^29^. In addition, prescribing patterns are likely to be influenced by other factors including, updates in guidelines recommendation. This was highlighted by Komen et al., who reported that the update in the European Guidelines was associated with an increase DOACs initiations^30^.

Our analysis also identified some of the individual-level characteristics that may influence the overall and the type of OAC prescribing. BMI ≥ 25 and male gender were strong predictors for the initiating of OAC. These results were also in line with a previous large observational study where the authors reported that both BMI ≥ 25 and male gender are likely to influence the OAC prescribing^31,32^. Other predictors including; the use of ACEI/ARB, BB, CCBs and statins were also associated with the initiation of OAC prescribing. This could be explained by the fact that these medications are commonly indicated for the management of cardiac diseases, where hypertension, peripheral vascular diseases, stroke and congestive heart failures are all criteria in CHA2DS2–VASc score calculations^33^. However, our results demonstrated that the use of aspirin was negatively associated with OAC prescribing. Aspirin is one of the criteria in HASBLED score^34^, in which it is given a total of 1 point in the total score which predicts the risk of bleeding and therefore, it reasonable to assume that patients who use aspirin are less likely to receive OAC. In addition, we found that both age ≥ 75 years and having a history of previous bleeding were significant predictors of DOAC prescribing. Several randomized trials studies have shown safer and non-inferiority of DOACs use in patients with AF^35–38^. In addition, recent observational studies have demonstrated a safer profile of DOACs compared to warfarin^39^, and less bleeding events among patients with AF ≥ 90 years of age^40^. Furthermore, having a history of CKD was associated with more likelihood of receiving DOACs. This finding was in line with some evidence-based literature, as DOACs showed favourable safety and efficacy profile in patients with CKD^41^.

### Strengths and limitations

To the best of our knowledge, this was the first study that examined the prevalence and treatment of AF in T2DM over a 16-years period. This study used a primary care database, which is representative of the UK general population, however, there are some limitations in our study. Firstly, THIN only provides information of primary care setting, and therefore, underestimation of the prevalence and treatment of AF in T2DM would be possible as THIN was not able to include patients from other health care settings. Secondly, patients were identified using relevant Read code lists and algorithms. In addition, we were not able to do data stratification based on AF type (i.e. paroxysmal, persistent, permanent) and management strategy (i.e. rhythm vs. rate control), which may influence OAC prescription rates.

## Conclusions

This study found that there was an increase in prevalence of AF in patients with T2DM between 2001 and 2016, and that both older and male patients were at higher risk of developing AF. The proportions of patients with T2DM who received OACs after AF diagnosis has increased during the study period. Further studies at individual and clinical practice level are warranted to investigate the factors associated with the underuse of OAC in patients with T2DM and AF in order to help in providing better responses and interventions in the management of this high-risk population.

## Supplementary information

Supplementary file1.

## Data Availability

No further data are available.

## References

[1] National Institute for Health and Care Excellence. Type 2 diabetes in adults: management.NICE guidline.[NG28] 2015 [Available from: https://www.nice.org.uk/guidance/ng28/resources/type-2-diabetes-in-adults-management-pdf-1837338615493. 26741015

[2] Nichols M, Townsend N, Scarborough P, Rayner M (2014). Cardiovascular disease in Europe 2014: epidemiological update. Eur. Heart J..

[3] Aune D, Feng T, Schlesinger S, Janszky I, Norat T, Riboli E (2018). Diabetes mellitus, blood glucose and the risk of atrial fibrillation: a systematic review and meta-analysis of cohort studies. J. Diabetes Complicat..

[4] Plitt A, McGuire DK, Giugliano RP (2017). Atrial fibrillation, Type 2 diabetes, and non-vitamin K antagonist oral anticoagulants: a review. JAMA Cardiol..

[5] Ogilvie IM, Newton N, Welner SA, Cowell W, Lip GY (2010). Underuse of oral anticoagulants in atrial fibrillation: a systematic review. Am J Med..

[6] National Institute for Health and Care Excellence. Atrial fibrillation management. NICE guideline (CG180) 2014. Available from: https://www.nice.org.uk/guidance/cg180. 31841281

[7] January Craig T, Wann LS, Calkins H, Chen Lin Y, Cigarroa Joaquin E, Cleveland Joseph C (2019). 2019 AHA/ACC/HRS focused update of the 2014 AHA/ACC/HRS guideline for the management of patients with atrial fibrillation: a report of the American College of Cardiology/American Heart Association Task Force on Clinical Practice Guidelines and the Heart Rhythm Society in Collaboration With the Society of Thoracic Surgeons. Circulation.

[8] Mekaj YH, Mekaj AY, Duci SB, Miftari EI (2015). New oral anticoagulants: their advantages and disadvantages compared with vitamin K antagonists in the prevention and treatment of patients with thromboembolic events. Ther. Clin. Risk Manag..

[9] Blak BT, Thompson M, Dattani H, Bourke A (2011). Generalisability of The Health Improvement Network (THIN) database: demographics, chronic disease prevalence and mortality rates. Inform. Primary Care..

[10] Banerjee A, Benedetto V, Gichuru P, Burnell J, Antoniou S, Schilling RJ (2020). Adherence and persistence to direct oral anticoagulants in atrial fibrillation: a population-based study. Heart (British Cardiac Society)..

[11] Brauer R, Lau WCY, Hayes JF, Man KKC, Osborn DPJ, Howard R (2019). Trazodone use and risk of dementia: a population-based cohort study. PLoS Med..

[12] Alwafi H, Wei L, Naser AY, Mongkhon P, Tse G, Man KKC (2020). Trends in oral anticoagulant prescribing in individuals with type 2 diabetes mellitus: a population-based study in the UK. BMJ Open..

[13] Fanning L, Lau WCY, Mongkhon P, Man KKC, Bell JS, Ilomäki J, et al. Safety and effectiveness of direct oral anticoagulants vs warfarin in people with atrial fibrillation and dementia. J. Am. Med. Dir. Assoc. 2020. 10.1016/j.jamda.2019.11.022 31917107

[14] Mongkhon P, Fanning L, Lau WCY, Tse G, Lau KK, Wei L (2020). Oral anticoagulant and reduced risk of dementia in patients with atrial fibrillation: a population-based cohort study. Heart Rhythm..

[15] Iwagami M, Tomlinson LA, Mansfield KE, Douglas IJ, Smeeth L, Nitsch D (2018). Gastrointestinal bleeding risk of selective serotonin reuptake inhibitors by level of kidney function: a population-based cohort study. Br. J. Clin. Pharmacol..

[16] Gieling EM, van den Ham HA, van Onzenoort H, Bos J, Kramers C, de Boer A (2017). Risk of major bleeding and stroke associated with the use of vitamin K antagonists, nonvitamin K antagonist oral anticoagulants and aspirin in patients with atrial fibrillation: a cohort study. Br. J. Clin. Pharmacol..

[17] Payne RA, Avery AJ (2011). Polypharmacy: one of the greatest prescribing challenges in general practice. Br. J. Gen. Pract.: J. R. Coll. Gen. Pract..

[18] Wagner AK, Soumerai SB, Zhang F, Ross-Degnan D (2002). Segmented regression analysis of interrupted time series studies in medication use research. J. Clin. Pharm. Ther..

[19] National Institute for Health and Care Excellence. Dabigatran etexilate for the prevention of stroke and systemic embolism in atrial fibrillation. Available from: https://www.google.com/url?sa=t&rct=j&q=&esrc=s&source=web&cd=1&ved=2ahUKEwjkvf64jMfnAhWAQxUIHVlBCDgQFjAAegQIBBAB&url=https%3A%2F%2Fwww.nice.org.uk%2Fguidance%2Fta249%2Fdocuments%2Fatrial-fibrillation-dabigatran-etexilate-final-appraisal-determination3&usg=AOvVaw3SSlCzafKjeBDCXcaH8TaS.

[20] Adderley NJ, Ryan R, Nirantharakumar K, Marshall T (2019). Prevalence and treatment of atrial fibrillation in UK general practice from 2000 to 2016. Heart (British Cardiac Society)..

[21] Welton NJ, McAleenan A, Thom HH, Davies P, Hollingworth W, Higgins JP (2017). Screening strategies for atrial fibrillation: a systematic review and cost-effectiveness analysis. Health Technol. Assess. (Winchester, England)..

[22] Wolf PA, Abbott RD, Kannel WB (1991). Atrial fibrillation as an independent risk factor for stroke: the Framingham Study. Stroke.

[23] Magnussen C, Niiranen TJ, Ojeda FM, Gianfagna F, Blankenberg S, Njolstad I (2017). Sex differences and similarities in atrial fibrillation epidemiology, risk factors, and mortality in community cohorts: results from the BiomarCaRE consortium (Biomarker for Cardiovascular Risk Assessment in Europe). Circulation.

[24] Goudis CA, Korantzopoulos P, Ntalas IV, Kallergis EM, Liu T, Ketikoglou DG (2015). Diabetes mellitus and atrial fibrillation: pathophysiological mechanisms and potential upstream therapies. Int. J. Cardiol..

[25] Bohne LJ, Johnson D, Rose RA, Wilton SB, Gillis AM. The Association between diabetes mellitus and atrial fibrillation: clinical and mechanistic insights. 2019;10(135). 10.3389/fphys.2019.00135 PMC6399657 30863315

[26] Gallagher C, Middeldorp ME, Sanders P (2019). Weight and risk of incident atrial fibrillation-body mass index variability or body mass gain?. Mayo Clin. Proc..

[27] Benjamin EJ, Levy D, Vaziri SM, D'Agostino RB, Belanger AJ, Wolf PA (1994). Independent risk factors for atrial fibrillation in a population-based cohort The Framingham Heart Study. JAMA.

[28] Kirchhof P, Benussi S, Kotecha D, Ahlsson A, Atar D, Casadei B (2016). 2016 ESC guidelines for the management of atrial fibrillation developed in collaboration with EACTS. Eur. Heart J..

[29] Lublóy Á (2014). Factors affecting the uptake of new medicines: a systematic literature review. BMC Health Serv. Res..

[30] Komen J, Forslund T, Hjemdahl P, Andersen M, Wettermark B (2017). Effects of policy interventions on the introduction of novel oral anticoagulants in Stockholm: an interrupted time series analysis. Br. J. Clin. Pharmacol..

[31] Rose AJ, Goldberg R, McManus DD, Kapoor A, Wang V, Liu W (2019). Anticoagulant prescribing for non-valvular atrial fibrillation in the veterans health administration. J. Am. Heart Assoc..

[32] Katz David F, Maddox Thomas M, Turakhia M, Gehi A, O’Brien Emily C, Lubitz Steven A, et al. Contemporary trends in oral anticoagulant prescription in atrial fibrillation patients at low to moderate risk of stroke after guideline-recommended change in use of the CHADS2 to the CHA2DS2-VASc score for thromboembolic risk assessment. Circ.: Cardiovasc. Qual. Outcomes. 2017;10(5):e003476. 10.1161/CIRCOUTCOMES.116.003476 28506981

[33] Lip GY, Nieuwlaat R, Pisters R, Lane DA, Crijns HJ (2010). Refining clinical risk stratification for predicting stroke and thromboembolism in atrial fibrillation using a novel risk factor-based approach: the euro heart survey on atrial fibrillation. Chest.

[34] Lane Deirdre A, Lip Gregory YH (2012). Use of the CHA2DS2-VASc and HAS-BLED scores to aid decision making for thromboprophylaxis in nonvalvular atrial fibrillation. Circulation.

[35] England PH. Atrial fibrillation prevalence estimates in England 2017. Available from: https://www.gov.uk/government/publications/atrial-fibrillation-prevalence-estimates-for-local-populations.

[36] Granger CB, Alexander JH, McMurray JJ, Lopes RD, Hylek EM, Hanna M (2011). Apixaban versus warfarin in patients with atrial fibrillation. N. Engl. J. Med..

[37] Patel MR, Mahaffey KW, Garg J, Pan G, Singer DE, Hacke W (2011). Rivaroxaban versus warfarin in nonvalvular atrial fibrillation. N. Engl. J. Med..

[38] Connolly SJ, Ezekowitz MD, Yusuf S, Eikelboom J, Oldgren J, Parekh A (2009). Dabigatran versus warfarin in patients with atrial fibrillation. N. Engl. J. Med..

[39] Vinogradova Y, Coupland C, Hill T, Hippisley-Cox J (2018). Risks and benefits of direct oral anticoagulants versus warfarin in a real world setting: cohort study in primary care. BMJ.

[40] Chao TF, Liu CJ, Lin YJ, Chang SL, Lo LW, Hu YF (2018). Oral anticoagulation in very elderly patients with atrial fibrillation: a nationwide cohort study. Circulation.

[41] Malhotra K, Ishfaq MF, Goyal N, Katsanos AH, Parissis J, Alexandrov AW (2019). Oral anticoagulation in patients with chronic kidney disease: a systematic review and meta-analysis. Neurology..

