# Supplementary material for: Epidemiology and treatment of atrial fibrillation in patients with type 2 diabetes in the UK, 2001–2016
Source: Sci Rep. 2020 Jul 27;10:12468. doi: 10.1038/s41598-020-69492-z (PMC7385086; doi:10.1038/s41598-020-69492-z)
Supplement: Supplementary file 1 — Supplementary file1. [file 41598_2020_69492_MOESM1_ESM.pdf]

SUPPLEMENTAL MATERIAL

Full title: Epidemiology and Treatment of Atrial Fibrillation in Patients with Type 2 Diabetes in the UK, 2001-2016.

Hassan Alwafi, MD[1,2], Ian CK Wong, PhD[1,3], Amitava Banerjee, DPhil[4,5], Pajaree Mongkhon, PhD[6,7], Cate Whittlesea, PhD[1], Abdallah Y Naser, PhD[8], Wallis C.Y. Lau, PhD[1,3], Li Wei, PhD[1]

[1]Research Department of Practice and Policy, School of Pharmacy, University College London, London, United Kingdom

[2]Faculty of Medicine, Umm Al Qura University, Mecca, Saudi Arabia

[3]Centre for Safe Medication Practice and Research, Department of Pharmacology and Pharmacy, Li Ka Shing Faculty of Medicine, The University of Hong Kong, Hong Kong

[4]Institute of Health Informatics, University College London, London, UK

[5]Barts Health NHS Trust, London, UK

[6]Division of Pharmacy Practice, Department of Pharmaceutical Care, School of Pharmaceutical Sciences, University of Phayao, Phayao, Thailand

[7]Pharmacoepidemiology and Statistics Research Center (PESRC), Faculty of Pharmacy, Chiang Mai University, Chiang Mai, Thailand

[8]Faculty of Pharmacy, Isra University, Amman, Jordan

Corresponding author
Professor Li Wei
UCL School of Pharmacy
29-39 Brunswick Square
London, WC1N 1AX
T: 020 7874 1275
E: l.wei@ucl.ac.uk

Tables (1)

Table S1**.** Interrupted time series analysis model on the changes before and after the introduction of DOACs in OAC prescribing

Table S1: Interrupted time series analysis model on the changes before and after the introduction of DOACs in OAC prescribing**.**

**Variable**	**IRR (95%CI)**	**Standard Error**	**P-value**
Change in level (immediate effect)	1.00 (0.99-1.01)	0.056	0.299
Change in trend post intervention	1.02 (1.01-1.03)	0.004	<.0001

Abbreviations: CI, confidence interval; IRR, incidence rate ratio;
